# Three-step checklist for tracheostomy in critically ill COVID-19 patients

**DOI:** 10.1186/s13054-020-03038-7

**Published:** 2020-06-08

**Authors:** Maria Vargas, Giuseppe Servillo

**Affiliations:** Department of Neurosciences, Reproductive and Odontostomatological Sciences, University of Naples “Federico II”, via Pansini, Naples, Italy

Tracheostomy is a common procedure in critically ill patients requiring prolonged mechanical ventilation [1]. The use of tracheostomy can facilitate weaning from ventilation and potentially increase the availability of intensive care unit (ICU) beds [2]. When the COVID-19 pandemic spread all around the world, ICUs had a massive influx of critically ill patients, many of whom became candidates for tracheostomy [2]. Tracheostomy is an aerosol-generating procedure that exposes physicians at high risk to contract infections [3]. In COVID-19 patients, healthcare workers who do tracheostomies must take into account additional considerations associated with the infectivity of SARS-CoV-2 [4]. Recent reports suggested to perform surgical and percutaneous tracheostomies with modified techniques to minimize the aerosol and then to keep the personnel safe [5, 6]. Although performing tracheostomy in COVID-19 patients is a high-acuity setting [6]. With such broad recognition of the importance of safety, we propose a three-step checklist to optimize the process of performing tracheostomy in critically ill COVID-19 patients (Fig. 1). The three-step checklist for tracheostomy in COVID-19 patients involves a preparation phase, a procedural phase, and an evaluation phase at the end of the procedure (Fig. 1). The preparation phase is intended to optimize all the action to prepare the patient and the staff for the procedure. The procedural phase includes the operative steps to perform the procedure with additional safety while the evaluation phase is intended to check the patient at the end of tracheostomy. Key points of this three-step checklist are proper wearing of personal protective equipment and actions to reduce the risk of viral aerosolization like pushed down the endotracheal tube and keep it cuffed during the procedure. We used the three-step checklist for tracheostomy in 3 percutaneous technique and 2 surgical techniques performed in critically ill COVID-19 patients, and we found that it is beneficial in preventing errors and harms. The three-step checklist for tracheostomy in critically ill COVID-19 patients is tailor-made to improve the safety and efficiency of a high-risk procedure for healthcare works.

**Fig. 1:**
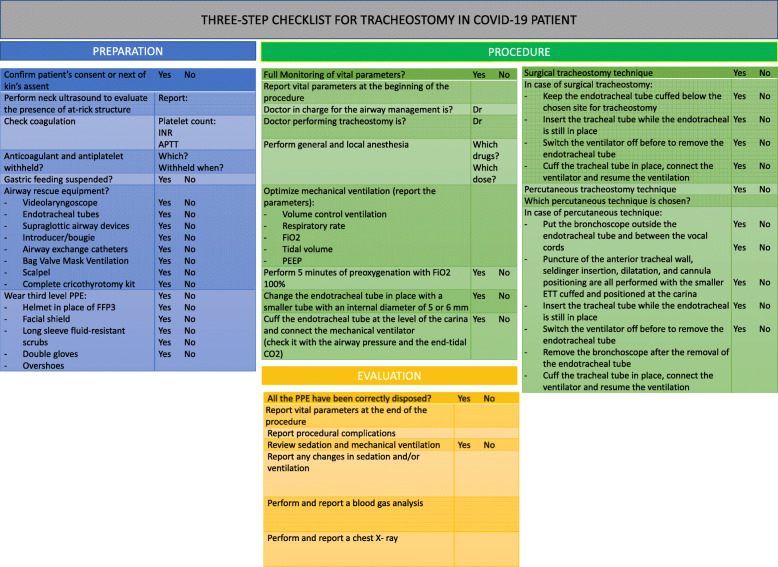
Three-step checklist for tracheostomy in critically ill COVID-19 patients

## Data Availability

NA
